# Physical properties, chemical composition, and nutritional evaluation of common salad dressings

**DOI:** 10.3389/fnut.2022.978648

**Published:** 2022-08-31

**Authors:** Mingyu Yin, Min Chen, Takuya Yanagisawa, Ryosuke Matsuoka, Yinci Xi, Ningping Tao, Xichang Wang

**Affiliations:** ^1^College of Food Science and Technology, Shanghai Ocean University, Shanghai, China; ^2^Food Science and Quality Evaluation Research Lab, Shanghai Ocean University, Shanghai, China

**Keywords:** salad dressing, lipids, fatty acid, minerals, nutritional evaluation, amino acids

## Abstract

Salad dressings (SDs), a subcategory of flavored sauces with more than 20% fat content and less than 30% moisture content, is favored by consumers due to its delicacy. The physical properties, chemical composition and nutritional evaluation of common SDs on the Chinese market needs to be systematically investigated. This study compared the quality (physical properties, proximate composition, amino acids, fatty acids, and minerals) of six commercially available sweet SDs (SD1, SD2, SD3, SD4, SD5, and SD6) from the Chinese market. The results indicated that the water activity of six SDs was less than 0.60 (0.35–0.41), the moisture content was less than 65% (24.0–60.0%), and the crude fat content was between 23.25 and 64.15%. The essential amino acid index (EAAI) of SD3, SD1, SD2, and SD4 was greater than the FAO/WHO standard (EAAI = 75). Numerous fatty acids were detected, mainly linoleic and oleic acids; n-3 polyunsaturated fatty acids were ranged from 1,090 mg/100 g to 2,520 mg/100 g. In addition, SDs were rich in minerals such as Mg, Ca, and Fe and the atherogenic index and thrombogenic index were 0.03–0.09 and 0.77–0.91, respectively. In summary, this work helps to provide key nutritional information on the composition of common SDs. The availability of this data may help purchasers with different nutritional needs to make informed choices about the use of SD and encourage more moderate consumption of pre-packaged sauces.

## Introduction

Salad dressing (SD) is a semisolid emulsion that originated on the Mediterranean island of Minorca. SD was invented in France in 1756 by the Duc de Richelieu’s Prefect (1). In 1905, Richard Herman’s New York delicatessen sold the first ready-made SD. SD is the most frequently consumed condiment in the United States, with domestic consumers spending approximately two billion dollars on the product (2). In China, SD production is still in its infancy, which means that salad dressing development will have a brighter future. SD is a creamy, thick sauce or condiment made with oil, egg yolks, lemon juice or vinegar, and seasonings.

Water, protein, fat, carbohydrates, minerals, and vitamins are the main substances in food (3). Vegetable oil (soybean oil, corn oil, etc.) and protein in SD are the main ingredient. Eggs, a critical component of SD, serve as an emulsifier, stabilizer, flavoring agent, and colorant (4). Egg yolks are a good source of nutrients because they contain triglycerides, phospholipids, stearidonic acid, fat-soluble vitamins (A and E), and essential fatty acids (5). Vegetable oil is an important raw material for SD, mainly containing vitamin E, vitamin K, calcium, iron, phosphorus, potassium and other minerals, fatty acids, etc., (2). The fatty acids in vegetable oils make the skin moist and shiny (6). The atherogenic index and thrombogenic index are important measures for assessing the nutritional level of lipids (7). The Chinese Nutrition Society recommends that 20 to 25% of the total energy provided by fats in the daily diet of adults is appropriate. As a component of the SD composition, acetic acid inhibits fermentation and spoilage, extends the shelf life of the SD, and imparts or enhances a distinctive flavor to the SD (8, 9). SD is frequently added to vegetables, sandwiches, and burgers as a condiment. Adjusting pH value and water activity value can ensure the shelf life of SD, color and sugar content will affect consumers’ sensory evaluation and stimulate their desire to consume (3). (10) used pea protein isolate in an SD-like Pickering emulsion and discovered that pea protein isolate microgels stabilized the SD-like Pickering emulsion. Several studies have shown that antioxidants can extend the shelf life of SD by retarding fat oxidation during storage (11, 12). It is necessary to conduct an analysis of the physical properties and basic composition of SD as a traded commodity, as well as a systematic nutritional and heavy metal risk assessment.

This study aims to compare the physical properties, proximate composition, fatty acid, amino acid, and mineral content of six popular sweet salad dressings (SDs) preferred by Chinese consumers. To calculate the risk index, a comprehensive nutritional assessment was conducted from three perspectives: proteins, fats, and minerals. This study systematically give the composition and nutritional evaluation of SDs on the market, providing data and a theoretical basis for consumer consumption and the development of functional condiments.

## Materials and methods

### Materials and reagents

Six different commercially available SDs (10 bottles per brand) were purchased from Shanghai’s Nonggongshang Supermarkets in May 2020. Samples were immediately taken to the laboratory and stored in a refrigerator at 4°C for further analysis, and coded (SD1, SD2, SD3, SD4, SD5, and SD6). The ingredient list for the samples were showed in Table 1.

**TABLE 1:** Information on salad dressing samples.

Samples	Production place	Ingredient list
SD1	Dongguan City, Guangdong Province, China	Water, soybean oil, Fructose Syrup, Whole Egg, Xanthan Gum, Glacial Acetic Acid, Lactic Acid, Phosphoric Acid, Potassium Sorbate, Sodium Dehydroacetate, Beta-Carotene, Sodium EDTA, Sucrose, Sodium Chloride
SD2	Qingdao City, Shandong Province, China	Water, soybean oil, Fructose Syrup, Whole Egg, Sucrose, Glacial acetic acid, sodium chloride, mustard powder, flavoring, monosodium glutamate, xanthan gum, disodium EDTA, steviol glycosides, beta-carotene, capsanthin
SD3	Tianjin, China	Water, Soybean Oil, Sugar, Whole Egg, Hydroxypropyl Distarch Phosphate, Sodium Chloride, Acetic Acid, Phosphoric Acid, Xanthan Gum, Disodium EDTA, Potassium Sorbate, Flavoring
SD4	Hangzhou City, Zhejiang Province, China	Water, Soybean oil, sugar, egg yolk, vinegar, sodium chloride, sodium glutamate, xanthan gum, steviol glycosides, disodium EDTA, lemon juice
SD5	Yunfu City, Guangdong Province, China	Water, soybean oil, sugar, sodium chloride, acetic acid, whole egg, hydroxypropyl distarch phosphate, xanthan gum, citric acid, beta-carotene, steviol glycosides, sodium benzoate, potassium sorbate, ethylenediamine disodium tetraacetate
SD6	Guangzhou City, Guangdong Province, China	Water, Soybean Oil, Sugar, Egg Yolk, Acetic Acid, Hydroxypropyl Distarch Phosphate, Xanthan Gum, Citric Acid, Potassium Sorbate, Sodium Benzoate, Beta-Carotene, Disodium EDTA, Sodium Chloride

Thirty-seven component standard fatty acid methyl ester (FAME), C19:0 fatty acid standard product, and C19:0 fatty acid methyl ester standard products were purchased from Yuan Ye Bio-Technology Co., Ltd., (Shanghai, China). Furthermore, the other reagents involved in this experiment were purchased from Sinopharm Chemical Reagent Co., Ltd., (Shanghai, China).

### Analysis of physical properties

#### Color difference

The 2.0 g sample was placed in an uncovered transparent cylindrical mold with 4-cm diameter and 2-cm height, the colorimeter was placed vertically on the surface of sample, and the L*, a*, and b* values were quickly determined. Whiteness is the degree of whiteness on the surface of the material, expressed as a percentage of white content, which is used to characterize the color acceptance of salad dressings. Each sample was measured six times in parallel.


(1) Whiteness=100-(100-L*)2+a*2+b*2

#### pH value, water activity, and conductivity

pH value was determined using a previously described method (10). The 2.00 g sample was accurately weighed and combined with 18 mL of distilled water, homogenized for 1 min, and centrifuged for 10 min at 12,000 r/min. pH was determined using a PHS-3C pH meter (Mettler Toledo, Zurich, Switzerland).

Conductivity was determined using a previously described method (13, 14). The 2.0 g sample was weighed precisely and dissolved in 18 mL of ultrapure water. The sample was homogenized for 1 min and centrifuged at 12,000 r/min for 10 min. The supernatant was collected, and its conductivity was determined using a conductivity meter (DDBJ-350, Qi Wei Instrument Co., Shanghai, China).

The water activity (Aw) of the samples was determined using instrumental measurements (Aqualab 4T, Decagon Devices, Pullman, WA, United States). The 1.0 g samples was precisely weighed and spread in the water activity meter’s special measuring container. Six parallel samples were collected.

#### Proximate composition analysis

Proximate composition of each sample was conducted using the method outlined by the Association of Official Analytical Chemists (15). Moisture content was determined by direct drying at a temperature of 105°C (AOAC 925.40). The crude fat and crude protein contents were determined using the Kjeldahl method (VELP UDK169) and the Soxhlet extraction method (AOAC 2003.05; AOAC 920.152), respectively. The ash content was determinted by the methods [AOAC (16)]. The results were given in grams per 100°grams of wet weight.

#### Fatty acid analysis

Fatty acid composition was determined using a previously described method (17). Briefly, 8.0 g of SD was mixed with 160 mL of CHCl_3_:CH_3_OH solution (2:1, V: V), and kept at 4°C for 24 h. Then, the solution was mixed with 30 mL of 0.9% NaCl, the lower layer was removed and concentrated under vacuum to obtain the total fat. The 5 mL 0.5 mol/mL NaOH-CH_3_OH was added to the 0.1 g fat. The mixture was placed on condensing and concentrating equipment (HWS24, HongLang, Henan, China) and heated at 100°C for 10 min. Subsequently, 3 mL boron trifluoride–methanol (14% in methanol) was added at 100°C and stirred for 3 min, followed by the addition of 2 mL n-hexane and maintaining at 100°C for 2 min. Finally, 10 mL saturated NaCl solution was added to the mixture. Samples were cooled to room temperature (24°C); the upper n-hexane layer was collected using a 2 mL disposable syringe, purified with a nylon syringe filter (13 mm × 0.22°μm), and stored in a 2 mL thread screw neck. Fatty acid composition was determined using a gas chromatograph (Trace GC Ultra, Thermo Fisher Scientific Inc., MA, United States) with a flame ionization detector (Thermo Fisher Scientific Inc.) equipped with an Agilent (Santa Clara, CA, United States) SP-2560 capillary column (100°m × 250°μm × 0.2°μm). The internal standard was C19:0.

#### Amino acid analysis

Amino acid content was determined using the method specified in (18). Briefly, 0.60 g of SD and 10 mL of 12 mol/L hydrochloric acid were placed in a 30 mL brown digestion tube and placed in an oven at 110°C for 22°h. Following the filtration of the residue, 1.0 mL of the filtrate was transferred to a tube and dried in a vacuum oven at 45°C. Next, the dried sample was drained three times with deionized water, 2 mL of citric acid solution was added, and the sample was stored for analysis.

The hydrolyzed samples were separated using an Inertsil ODS-3 C18 column (4.6 mm × 150 mm, 7°μm, GL Sciences Inc., Tokyo, Japan), and amino acid composition was determined using the liquid chromatography (L-8800, Hitachi Co., Ltd., Tokyo, Japan). The column was Inertsil ODS-3 C18 (4.6 mm × 150 mm, 7°μm, GL Sciences Inc., Tokyo, Japan). The mobile phase was a mixed buffer of sodium citrate and citric acid with pH 3.2, 3.3, 4.0, and 4.9, and a ninhydrin buffer with a mass fraction of 4%.

#### Mineral analysis

Here, 0.50 g of SD was mixed with 5.0 mL of 68% HNO_3_ solution, was sealed in a digestion tube, and was left to stand for 24 h before being digested (the procedure is as follows: 0–5 min, sample temperature was raised to 120°C and held for 5 min, raised to 160°C at an 8°C/min rate and held for 5 min, and raised to 190°C at an 8°C/min rate and held for 20 min). After cooling the digestion tube, it was heated to 120°C for 30 min and then diluted to 50 mL with ultrapure water. Seven target minerals (Na, K, Mg, Ca, Zn, Fe, and Se) were analyzed using a quadrupole inductively coupled plasma mass spectrometer (ICP-MS, iCAP-Qc, United States). ^73^Ge, ^89^Y, ^115^In, and ^209^Bi were used as internal standards.

### Nutritional evaluation

The atherogenic index (AI) and thrombogenicity index (TI), important parameters for assessing the health value of fatty acids, were calculated to assess the nutritional quality of FA. Each of these was calculated as follows:


(2) $$\text{AI}=\frac{{\text{C}}_{12:0}+4\,{\text{C}}_{14:0}+{\text{C}}_{16:0}}{\sum \text{MUFA}+\sum \text{PUFA}},$$

(3) $$\text{TI}=\frac{\begin{array}{c} {\text{C}}_{14:0}+{\text{C}}_{16:0}+{\text{C}}_{18:0} \end{array}}{\begin{array}{c} 0.5\,\sum \text{MUFA}+0.5\,\sum \text{n}-6\,P\,U\,F\,A+3\,\sum \text{n}-3\,P\,U\,F\,A\\ +\frac{\sum \text{n}-3\,P\,U\,F\,A}{\sum \text{n}-6\,P\,U\,F\,A},\left(2\right) \end{array}}$$

where ΣMUFA was the total amount of monounsaturated fatty acids, ΣPUFA was the total amount of polyunsaturated fatty acids, Σn-3 PUFA was the total amount of n-3 polyunsaturated fatty acids, and Σn-6 PUFA was the total amount of n-6 polyunsaturated fatty acids.

Amino acid composition was determined using the essential amino acid index (EAAI). The relative nutrition index was calculated using the formula provided by the FAO/WHO [FAO/WHO, (19)]. Furthermore, the egg protein model proposed by the Chinese Academy of Preventive Medicine’s Institute of Nutrition and Food Hygiene for nutritional evaluation was used to calculate the amino acid score (AAS), chemical score (CS), and amino acid ratio coefficient (RC) in accordance with the Dietary Protein Quality Evaluation in Human Nutrition (20) as follows:


(4) $$\text{EAAI}=\sqrt[{\text{n}}]{\frac{{\text{T}}_{\text{Lys}}}{{\text{S}}_{\text{Lys}}}\times 100\times \frac{{\text{T}}_{\text{Leu}}}{{\text{S}}_{\text{Leu}}}\times 100\times \cdots \times \frac{{\text{T}}_{\text{Val}}}{{\text{S}}_{\text{Val}}}\times 100}$$

(5) $$\text{AAS}=\frac{\begin{array}{c} \mathrm{Content}\,\mathrm{of}\,\mathrm{essential}\,\mathrm{amino}\,\mathrm{acids}\,\mathrm{in}\,\mathrm{the}\\ \mathrm{sample}\,\left(\mathrm{mg}/100\,g\,\mathrm{protein}\right) \end{array}}{\begin{array}{c} \mathrm{Content}\,\mathrm{of}\,\mathrm{essential}\,\mathrm{amino}\,\mathrm{acids}\,\mathrm{in}\,\mathrm{the}\\ \mathrm{standard}\,\mathrm{model}\,\left(\mathrm{mg}/100\,g\,\mathrm{protein}\right) \end{array}}\times 100$$

(6) $$\text{CS}=\frac{\begin{array}{c} \mathrm{Content}\,\mathrm{of}\,\mathrm{essential}\,\mathrm{amino}\,\mathrm{acids}\,\mathrm{in}\,\mathrm{the}\\ \mathrm{sample}\,\left(\mathrm{mg}/100\,g\,\mathrm{protein}\right) \end{array}}{\begin{array}{c} \mathrm{Content}\,\mathrm{of}\,\mathrm{essential}\,\mathrm{amino}\,\mathrm{acids}\,\mathrm{in}\,\mathrm{the}\\ \mathrm{Egg}\,\mathrm{standard}\,\mathrm{model}\,\left(\mathrm{mg}/100\,g\,\mathrm{protein}\right) \end{array}}\times 100$$

(7) $$\text{RC}=\frac{\mathrm{Scoring}\,\mathrm{of}\,\mathrm{AAS}\,\mathrm{in}\,\mathrm{tested}\,\mathrm{samples}}{\begin{array}{c} \mathrm{Mean}\,\mathrm{of}\,\mathrm{essential}\,\mathrm{amino}\,\mathrm{acids}\,\left(\mathrm{EAA}\right)\\ \mathrm{score}\,\mathrm{values}\,\mathrm{among}\,\mathrm{tested}\,\mathrm{food}\,\mathrm{proteins} \end{array}}\times 100\%$$

where the standard model protein was determined according to the FAO/WHO standard mode, n denoted the number of EAA compared, T denoted the amino acid content of the sample (mg/100 g protein), and S denotes the amino acid content of egg protein (mg/100 g protein).

### Statistical analysis

Data was expressed as mean ± standard error of the mean (SEM). SPSS 21.0 software was used to analyze all data collected in this study. The difference in samples was analyzed using one-way analysis of variance (ANOVA) and Duncan’s test at the level of significance *P* < 0.05.

## Results and discussion

### Physical properties of salad dressings

The physical properties of six commercially available sweetened SDs were summarized in Table 2. The pH of SD should be less than the recommended value of 4.0 for long-term storage (4). The pH of different SDs were ranked as follows: SD2 > SD6 > SD4 > SD3 > SD1 > SD5. The pH values of the SDs differed, with the lowest in the SD5 group (pH = 3.34 ± 0.04) and the highest in the SD2 group (pH = 3.99 ± 0.03). Conductivity was used to describe the ionic level in an indirect manner (2). The conductivity in SD1, SD3, and SD6 was significantly higher than the SD2, SD4, and SD5 groups (*P* < 0.05). Aw was significantly correlated with food safety and stability. The Aw of SDs was below 0.60 (SD1: 0.378 ± 0.006; SD2: 0.371 ± 0.002; SD3: 0.372 ± 0.002; SD4: 0.37 ± 0.001; SD5: 0.407 ± 0.005; and SD6: 0.37 ± 0.001), indicating that the product was considered relatively safe (21). The SD2 had the highest L* and b* values, representing brighter and yellowish, implying that SD2 was more acceptable to consumers than the other groups. The values for whiteness revealed that the SD6 group had significantly higher values compared to the other five groups (*P* < 0.05) and the SD1 group had the lowest values. The SDs were pale yellow, which might be due to the use of egg yolk in these SDs (22). The SD prepared by Song et al. (1) had lower L* value, higher b* values, and lower sensory scores, which was consistent with our results.

**TABLE 2:** Analysis of the physical properties of different sweet salad dressings.

	SD1	SD2	SD3	SD4	SD5	SD6
pH	3.52 ± 0.02^d^	3.99 ± 0.03^a^	3.67 ± 0.01^c^	3.91 ± 0.02^b^	3.34 ± 0.04^e^	3.96 ± 0.04^a^
Electrical conductivity	4829.75 ± 3.40^a^	2983.50 ± 1.00^d^	4693.00 ± 3.56^b^	2975.00 ± 1.63^d^	2444.75 ± 4.27^e^	4127.00 ± 15.75^c^
Water activity	0.378 ± 0.006^b^	0.371 ± 0.002^c^	0.372 ± 0.002^bc^	0.37 ± 0.001^c^	0.407 ± 0.005^a^	0.37 ± 0.001^c^
Sugar content (%)	33.78 ± 0.90^e^	41.60 ± 1.29^ab^	42.42 ± 0.43^a^	37.72 ± 0.29^d^	38.96 ± 0.44^c^	40.80 ± 0.55^b^
L*	68.18 ± 1.04^b^	74.61 ± 4.06^a^	66.56 ± 1.00^b^	66.78 ± 0.70^b^	67.65 ± 0.94^b^	62.07 ± 0.72^c^
a*	–2.88 ± 0.05^c^	–2.77 ± 0.14^b^	–3.23 ± 0.10^d^	–3.23 ± 0.10^d^	–2.21 ± 0.09^a^	–2.96 ± 0.04^c^
b*	10.40 ± 0.09^c^	13.60 ± 0.56^a^	9.59 ± 0.09^d^	10.70 ± 0.15^c^	7.66 ± 0.13^e^	12.29 ± 0.18^b^
Whiteness	30.97 ± 1.02^b^	24.11 ± 4.05^c^	32.68 ± 1.01^b^	32.29 ± 0.71^b^	31.88 ± 0.93^b^	36.65 ± 0.70^a^

Data are presented as mean ± SEM (n = 6). Different letters indicate significant differences at P < 0.05 determined by ANOVA (Duncan’s test). L* represented lightness; a* represented redness/greenness; b* represented yellowness/blueness.

### Proximate composition analysis

The approximate composition of the six SDs were shown in Table 3. The moisture content of SDs were as follows: SD1 (51.94 ± 0.19 g/100 g) > SD5 (45.18 ± 0.72 g/100 g) > SD3 (45.15 ± 0.45 g/100 g) > SD6 (41.89 ± 1.05 g/100 g) > SD2 (24.79 ± 0.22 g/100 g) > SD4 (21.58 ± 0.26 g/100 g). The crude fat content in SDs differed from the results for moisture content, which was consistent with previous studies (23). Furthermore, SDs had a crude fat content from 23.25 to 64.15 g/100 g, which classified as a high-fat food (12). This was consistent with the 16.63–59.93 g/100 g moisture content and 13.72–53.74 g/100 g crude fat content of SD in the Malaysian market (24). In addition, SD3 had a significantly higher crude protein content compared to SD1, SD2, SD4, SD5, and SD6 (*P* < 0.05). Similarly, SD1 had a significantly higher ash content compared to SD4 and SD5 (*P* < 0.05), but was no significant differences with SD2, SD3, and SD6.

**TABLE 3:** Proximate composition of different sweet salad dressings (g/100g).

	SD1	SD2	SD3	SD4	SD5	SD6
Moisture	51.94 ± 0.19^a^	24.79 ± 0.22^c^	45.15 ± 0.45^b^	21.58 ± 0.26^c^	45.18 ± 0.72^b^	41.89 ± 1.05^b^
Crude fat	23.25 ± 1.02^d^	63.15 ± 0.72^a^	29.15 ± 1.41^c^	64.15 ± 0.98^a^	33.07 ± 1.22^b^	44.07 ± 5.52^b^
Crude protein	0.16 ± 0.03^e^	0.37 ± 0.04^d^	0.88 ± 0.05^a^	0.71 ± 0.01^b^	0.51 ± 0.13^c^	0.70 ± 0.05^b^
Ash	2.63 ± 0.22^a^	1.84 ± 0.21^ab^	2.48 ± 0.13^a^	1.68 ± 0.39^b^	1.24 ± 0.04^b^	2.56 ± 0.56^a^

Data are presented as mean ± SEM (n = 5). Different letters indicate significant differences at P < 0.05 determined by ANOVA (Duncan’s test).

### Fatty acid analysis

Fatty acids are key cellular components, comprising parts of the cell membrane, organelles and cytosol. Unsaturated fatty acids (UFA) have significant antioxidant and antiaging properties, as well as obvious protective effects on the vascular barrier and permeability (6). The fatty acid composition of SDs were shown in Table 4. In all, 28 fatty acids were detected in SDs, including 13 SFAs, 6 MUFAs, and 9 PUFAs. The order of composition of the different saturated fatty acids was as follows.: UFA > PUFA > MUFA > SFA. SD2 had a significantly higher UFA content than the others (*P* < 0.05). Linoleic acid (C18:2, 35.44–51.37%) and oleic acid (C18:1, 18.29–24.04%) were the most abundant fatty acids in SD1, SD2, SD3, SD4, SD5, and SD6. The C18:2 has been shown to lower blood cholesterol and prevent atherosclerosis (25). The content of C18:2 in SD ranged from 89.16 to 265.56 mg/g, suggesting its hypolipidemic potential. Based on this comparison, the pathogenicity of salad dressings for cardiovascular disease has raised concerns.

**TABLE 4:** Fatty acid composition and nutrition score of lipids in different sweet salad dressings.

	Fatty acid (mg/100 g salad dressing)					
	SD1	SD2	SD3	SD4	SD5	SD6
C4:0	6.07 ± 3.01^ab^	11.88 ± 4.36^a^	1.99 ± 0.05^b^	8.5 ± 7.78^ab^	4.99 ± 1.66^ab^	4.16 ± 3.73^ab^
C6:0	1.13 ± 0.55^b^	1.22 ± 1.41^b^	0.33 ± 0.12^b^	1.91 ± 1.72^ab^	2.57 ± 0.77^ab^	3.71 ± 0.99^a^
C12:0	0.47 ± 0.36^c^	1.57 ± 0.21^a^	0.77 ± 0.21^bc^	0.50 ± 0.17^c^	0.86 ± 0.09^bc^	1.03 ± 0.36^b^
C14:0	13.34 ± 0.66^c^	37.31 ± 4.19^a^	12.8 ± 0.57^c^	43.04 ± 1.28^a^	23.78 ± 2.77^b^	26.92 ± 6.82^b^
C15:0	2.50 ± 0.16^c^	6.25 ± 0.30^a^	4.02 ± 1.33^bc^	7.06 ± 1.07^a^	4.14 ± 0.26^b^	4.97 ± 0.67^b^
C16:0	943.64 ± 29.90^d^	2744.24 ± 273.29^a^	1195.43 ± 37.13^d^	2930.14 ± 14.70^a^	1551.95 ± 109.50^c^	1936.04 ± 207.15^b^
C16:1	18.72 ± 1.32^d^	49.48 ± 7.37^b^	31.26 ± 1.12^c^	67.71 ± 2.37^a^	31.33 ± 6.70^c^	47.08 ± 4.80^b^
C17:0	17.63 ± 0.79^d^	49.02 ± 4.42^a^	29.23 ± 1.65^c^	51.47 ± 2.24^a^	28.08 ± 3.18^c^	37.81 ± 3.73^b^
C17:1	6.70 ± 4.66^d^	22.11 ± 0.93^a^	12.58 ± 0.57^c^	23.78 ± 0.91^a^	14.92 ± 2.53^b^	19.51 ± 3.80^ab^
C18:0	880.22 ± 29.24^d^	2129.21 ± 235.14^a^	1323.08 ± 7.47^c^	2169.26 ± 51.27^a^	1298.94 ± 98.96^c^	1674.38 ± 129.24^b^
C18:1	4287.11 ± 174.95^d^	10762.54 ± 1954.05^ab^	5516.14 ± 1310.63^d^	12338.98 ± 315.47^a^	7251.66 ± 746.38^c^	8097.42 ± 695.92^bc^
C18:2	8916.74 ± 391.6^d^	22681.62 ± 2874.7^ab^	13529.2 ± 3028.64^cd^	26556.74 ± 854.35^a^	14537.2 ± 1550.74^c^	19393.47 ± 1664.69^b^
C20:0	82.61 ± 2.21^cd^	231.29 ± 40.51^a^	79.57 ± 15.98^d^	198.59 ± 7.40^a^	125.19 ± 15.82^bc^	134.8 ± 11.54^b^
C20:1	54.12 ± 12.03^b^	104.66 ± 90.64^b^	53.84 ± 1.96^b^	395.29 ± 190.94^a^	140.91 ± 78.86^b^	222.47 ± 123.30^ab^
C18:3	1094.75 ± 45.51^b^	1771.63 ± 1549.82^ab^	1609.66 ± 570.37^ab^	2496.62 ± 92.52^a^	1473.8 ± 321.86^ab^	2225.44 ± 184.17^a^
C21:0	2.05 ± 1.37^b^	5.96 ± 6.95^b^	6.52 ± 5.47^b^	16.42 ± 1.52^a^	6.29 ± 2.35^b^	8.31 ± 1.24^b^
C20:2	5.16 ± 3.43^c^	27.21 ± 3.38^ab^	12.72 ± 4.95^bc^	30.62 ± 21.80^a^	16.89 ± 5.30*^abc^*	22.02 ± 1.87*^abc^*
C22:0	95.22 ± 1.75^d^	292.88 ± 40.60^a^	102.92 ± 3.64^cd^	230.52 ± 17.00^b^	140.78 ± 33.69^c^	142.32 ± 6.23^c^
C20:3	0.57 ± 0.53	1.24 ± 2.11	0.16 ± 0.14	0.22 ± 0.11	2.99 ± 2.81	3.1 ± 3.92
C22:1	0.46 ± 0.42^b^	18.78 ± 5.08^b^	0.77 ± 0.80^b^	1.45 ± 1.74	18.29 ± 28.08^b^	62.48 ± 4.94^a^
C20:3	0.05 ± 0.08	0.52 ± 0.60	0.36 ± 0.50	0.46 ± 0.53	0.44 ± 0.54	0.73 ± 0.70
C20:4	8.59 ± 0.55^d^	29.73 ± 1.10^a^	23.37 ± 1.86^b^	23.3 ± 2.59^b^	15.57 ± 3.06^c^	14.68 ± 2.12^c^
C23:0	0.36 ± 0.48^c^	6.54 ± 0.31^b^	2.45 ± 3.40^bc^	2.10 ± 2.07^bc^	5.66 ± 5.80^bc^	15.84 ± 1.13^a^
C22:2	0.17 ± 0.15	0.03 ± 0.04	0.04 ± 0.02	0.19 ± 0.08	0.20 ± 0.22	0.14 ± 0.15
C24:0	33.63 ± 5.54^e^	106.85 ± 3.08^a^	65.51 ± 6.88^bc^	77.3 ± 5.87^b^	54.45 ± 13.16^cd^	51.49 ± 4.56^d^
C20:5	0.38 ± 0.36^c^	2.16 ± 3.56^b^	1.19 ± 1.61^b^	6.49 ± 0.39^a^	0.06 ± 0.01^c^	1.21 ± 1.85^b^
C24:1	0.51 ± 0.97^b^	5.3 ± 6.53^ab^	7.79 ± 5.01^ab^	4.49 ± 6.95^ab^	9.54 ± 3.30^ab^	10.27 ± 4.45^a^
C22:6	0.03 ± 0.02^c^	1.72 ± 2.72^b^	0.06 ± 0.03^c^	8.13 ± 0.22^a^	0.23 ± 0.30^c^	0.35 ± 0.37^c^
EPA+DHA	0.4 ± 0.37^c^	3.89 ± 6.28^b^	1.25 ± 1.58^b^	14.62 ± 0.94^a^	0.29 ± 0.29^c^	1.56 ± 1.62^b^
ΣSFA	2079.16 ± 44.53^d^	5653.61 ± 590.25^a^	2751.02 ± 315.85^c^	5737.94 ± 81.60^a^	3248.68 ± 217.01^c^	4043.7 ± 356.15^b^
ΣMUFA	4368.97 ± 184.48^d^	10983.07 ± 2033.88^ab^	5682.91 ± 256.37^cd^	12842.94 ± 156.29^a^	7477.07 ± 679.33^c^	8469.57 ± 820.28^bc^
ΣPUFA	10094.85 ± 442.95^d^	24714.81 ± 4254^ab^	10039.59 ± 1384.37^d^	29578.32 ± 974.78^a^	16252.87 ± 1885.73^c^	21961.61 ± 1870.47^b^
n-3 PUFA	1095.87 ± 46.02^b^	1773.99 ± 1553.64^ab^	1610.73 ± 573.12^ab^	2509.94 ± 92.86^a^	1473.92 ± 321.93^ab^	2226.35 ± 186.03^a^
ΣUFA	14463.82 ± 626.58^e^	35697.89 ± 6287.56^ab^	15646.16 ± 1024.45^de^	42421.26 ± 1085.61^a^	23729.95 ± 2002.79^cd^	30431.18 ± 2689.76^bc^
ΣFA	16542.98 ± 642.11^e^	41351.49 ± 6481.75^ab^	18329.28 ± 1310.2^de^	48159.20 ± 1139.58^a^	26978.63 ± 2212.13^cd^	34474.88 ± 3045.87^bc^
Atherogenicity index	0.07 ± 0	0.08 ± 0.01	0.03 ± 0.03	0.07 ± 0	0.07 ± 0	0.07 ± 0
Thrombogenicity index	0.84 ± 0.04	0.91 ± 0.15	0.91 ± 0.18	0.8 ± 0	0.77 ± 0.07	0.86 ± 0

Data are presented as mean ± SEM (n = 3). Different letters indicate significant differences at P < 0.05 determined by ANOVA (Duncan’s test). EPA, eicosapentaenoic acid; DHA, docosahexaenoic acid.

Linoleic acid is an n-6 PUFA and oleic acid is a representative n-9 series fatty acid. These two fatty acids can provide energy to humans and regulates the ratio of high-density lipoprotein cholesterol (HDL-c) to low-density lipoprotein cholesterol (LDL-c) in plasma (26). Additionally, linolenic acid (C18:3), docosahexaenoic acid (DHA) and eicosapentaenoic acid (EPA), which are essential n-3 fatty acids, can help lower blood lipids and cholesterol levels (27), boost immune function (28), promote vision, and intellectual development (14). The content of C18:3 and n-3 polyunsaturated fatty acids (n-3 PUFA) in SD4 and SD6 was significantly higher than that in SD1, SD2, SD3, and SD5, suggesting that SD4 and SD6 might be products reducing cardiovascular disease. Compared to SD1, SD2, SD3, SD5, and SD6, There was approximately 8–14% SFA of total fatty acids (TFA) in six samples. A previous study found that the SFA content of hempseed oil (10.79%), moringa oil (22.74%), echium oil (10.98%), extra virgin olive oil (16.96%), and linseed oil (8.78%) (29), which was consistent with the results of this study. A previous study found that the SFA content of some traditional fish and shrimp paste condiments ranged between 46 and 73.14%, indicating that fatty acid composition of SDs was clearly superior to that of traditional fish and shrimp paste condiments (30). Consumers would decide to restrict their consumption of SDs based on the fat content, while this results showed that although SD contained SFA, it was a good source of biofunctional lipids.

The AI and TI can be used to determine the degree to which fatty acid composition is associated with cardiovascular disease (7). These indices have been linked to the diet-heart hypothesis, coronary artery disease, thrombosis, and the formation of atherosclerosis (31). The AI and TI values in SDs was 0.03–0.09 and 0.77–0.91, respectively, which were significantly lower than those of chicken meat (AI: 0.650–0.891) (32) and *Thunnus thynnus L.* (AI: 0.624–0.782) (33). The low AI and TI values indicate a low risk of causing cardiovascular disease. Therefore, moderate consumption SD is beneficial to human health, especially SD4, and SD5.

### Amino acid analysis

The Amino acids, the building blocks of proteins, are critical components of human nutrition and health. Amino acid is required for various physiological functions, including the regulation of human metabolism and neurological development (34). Table 5 summarizes the amino acid (AA) composition of six SDs. There were 18 AAs detected, including 7 EAAs and 9 nonessential amino acids (NEAA), indicating that SD is an excellent source of amino acids. The order of amino acids was SD3 > SD6 > SD4 > SD5 > SD2 > SD1, which was similar to the order of crude protein in SDs (SD3 > SD4 > SD6 > SD5 > SD2 > SD1). Glutamic acid (Glu) had the highest content, followed by leucine (Leu), aspartic acid (Asp), arginine (Arg), and alanine (Ala). The FAO/WHO ideal amino acid model [FAO/WHO, (19); FAO/WHO, (35)] indicates that the quality protein values of EAA/TAA are approximately 40%. The EAA/TAA ratios in SD1, SD2, SD3, SD4, SD5, and SD6 ranged from 35.99 to 42.00%. The SD1 and SD3 groups had an EAA/TAA ratio higher than 40.00% and appeared to be a better source of dietary protein. This result was comparable to the composition of quality protein values in other condiments [fish sauce: 38–46% (36); soybean: 46.6–52.9% (37)]. These findings indicated that the EAA/TAA values in SD1, SD2, SD3, SD4, SD5, and SD6 were comparable to those recommended. Additionally, SDs had a greater variety and concentration of amino acids compared to ketchup (another condiment) (38). As a result, it was clear that SD was a source of high-quality protein.

**TABLE 5:** Amino acid content in different sweet salad dressing (mg/100 g).

	SD1	SD2	SD3	SD4	SD5	SD6
Aspartic acid	21.15 ± 1.57^d^	31.98 ± 1.26^cd^	112.10 ± 2.53^a^	36.17 ± 1.27^c^	41.89 ± 17.74^bc^	52.56 ± 4.10^b^
Threonine*	10.22 ± 0.73^d^	18.81 ± 0.91^c^	52.92 ± 1.20^a^	24.21 ± 0.31^bc^	18.84 ± 7.90^c^	29.87 ± 2.15^b^
Serine	16.27 ± 1.19^d^	29.96 ± 1.27^c^	82.64 ± 1.74^a^	38.51 ± 0.48^bc^	28.79 ± 12.12^c^	45.36 ± 3.49^b^
Glutamic acid	22.75 ± 1.85^c^	87.45 ± 3.68^b^	128.30 ± 2.90^a^	93.60 ± 3.13^b^	90.13 ± 38.79^b^	66.29 ± 5.80^b^
Glycine	7.21 ± 0.47^d^	13.81 ± 0.4^c^	38.58 ± 0.70^a^	13.39 ± 0.38^c^	14.94 ± 6.47^c^	23.90 ± 1.80^b^
Alanine	13.92 ± 1.04^d^	22.17 ± 0.83^cd^	72.10 ± 1.34^a^	23.61 ± 0.60^c^	26.70 ± 11.62^bc^	34.81 ± 2.62^b^
Cystine	0.20 ± 0.06^c^	1.77 ± 0.09^b^	7.22 ± 0.23^a^	1.70 ± 0.21^b^	3.12 ± 1.86^b^	2.26 ± 0.18^b^
Valine*	14.40 ± 1.08^d^	24.04 ± 1.28^c^	63.82 ± 1.33^a^	27.48 ± 0.39^bc^	25.50 ± 10.26^c^	33.79 ± 2.28^b^
Methionine*	3.25 ± 0.25^d^	1.93 ± 0.02^e^	24.32 ± 0.25^a^	8.84 ± 0.10^b^	2.69 ± 1.23^de^	4.64 ± 0.13^c^
Isoleucine*	13.07 ± 0.90^d^	23.97 ± 1.19^c^	57.36 ± 1.24^a^	29.55 ± 0.20^bc^	22.83 ± 9.05^c^	33.16 ± 2.33^b^
Leucine*	24.88 ± 1.70^d^	39.46 ± 1.97^c^	107.65 ± 2.35^a^	47.55 ± 0.66^c^	42.63 ± 16.05^c^	60.05 ± 4.31^b^
Tyrosine	8.84 ± 0.77^d^	14.06 ± 0.85^c^	34.47 ± 0.99^a^	17.05 ± 0.18^bc^	15.74 ± 6.18^bc^	20.42 ± 1.52^b^
Phenylalanine*	12.32 ± 0.74^c^	20.21 ± 0.95^b^	55.71 ± 1.33^a^	20.57 ± 0.16^b^	24.17 ± 9.65^b^	27.20 ± 2.10^b^
Lysine*	9.28 ± 0.73^d^	17.11 ± 0.75^c^	49.08 ± 0.89^a^	21.76 ± 0.47^bc^	17.39 ± 7.60^c^	27.12 ± 2.07^b^
Histidine	1.93 ± 0.15^e^	3.96 ± 0.23^d^	10.28 ± 0.16^a^	7.94 ± 0.21^b^	3.80 ± 1.62^d^	6.26 ± 0.44^c^
Arginine	13.89 ± 1.03^d^	24.93 ± 1.49^c^	65.67 ± 1.61^a^	31.54 ± 0.51^c^	24.65 ± 10.55^c^	43.38 ± 3.20^b^
Proline	11.43 ± 0.65^d^	14.53 ± 0.90^cd^	50.54 ± 2.04^a^	15.04 ± 0.50^cd^	18.28 ± 6.69^c^	32.00 ± 2.11^b^
Hydroxyproline	N.D.	13.47 ± 0.97^b^	1.14 ± 0.14^cd^	29.88 ± 4.06^a^	3.94 ± 0.48^c^	1.80 ± 0.19^cd^
NEAA	117.60 ± 8.53	258.08 ± 11.77	603.03 ± 12.72	308.43 ± 10.07	271.97 ± 114.04	329.03 ± 25.39
EAA	87.42 ± 5.94	145.53 ± 7.02	410.87 ± 8.09	179.95 ± 2.00	154.05 ± 61.55	215.83 ± 15.27
TAA	206.38 ± 14.58	405.37 ± 18.82	1017.85 ± 19.96	490.45 ± 11.15	427.43 ± 176.28	548.17 ± 41.12
NEAA/TAA	0.57 ± 0.00	0.64 ± 0.01	0.59 ± 0.02	0.63 ± 0.01	0.63 ± 0.01	0.60 ± 0.02
EAA/TAA	0.42 ± 0.00	0.36 ± 0.01	0.40 ± 0.03	0.37 ± 0.01	0.36 ± 0.01	0.39 ± 0.01

Data are presented as mean ± SEM (n = 3).

Different letters denote significant differences at P < 0.05 determined by ANOVA (Duncan’s test). NEAA, nonessential amino acid; EAA, essential amino acid content; TAA, total amino acid content.

* Represented essential amino acid.

The nutritional score of SD was calculated using the WHO/FAO protein scoring model (1993). As shown in Table 6, a comprehensive analysis of the amino acid score (AAS) and chemical score (CS) revealed that Ala and Tyr, along with Lys, were the first and second limiting amino acids in the six samples, respectively.

**TABLE 6:** Comparative nutrition indices of different sweet salad dressings and the adult essential amino acid (EAA) model recommended by FAO/WHO (19).

		Threonine	Valine	Isoleucine	Leucine	Methionine + cystine^▲^	Phenylalanine + tyrosine	Lysine	EAAI
SD1	AAS	1.50	1.66	1.91	3.01	0.55	2.02	0.99	107.73
	CS	1.28	1.33	1.21	2.48	0.31	1.37	0.77	
	RC	0.90	1.00	1.14	1.80	0.33	1.21	0.59	
SD2	AAS	1.20	1.23	1.68	1.57	0.28	1.70	0.87	77.56
	CS	1.02	0.99	1.07	1.29	0.16	1.15	0.68	
	RC	0.99	1.02	1.39	1.30	0.23	1.40	0.72	
SD3	AAS	0.93	0.85	1.82	1.04	0.46	0.93	0.62	103.68
	CS	0.79	0.68	1.16	0.85	0.26	0.63	0.48	
	RC	1.00	0.92	1.96	1.11	0.49	1.00	0.66	
SD4	AAS	1.16	1.26	1.36	1.43	0.39	0.70	0.79	79.43
	CS	0.99	1.01	0.86	1.18	0.22	0.47	0.61	
	RC	1.15	1.25	1.34	1.42	0.39	0.69	0.78	
SD5	AAS	1.13	1.03	1.09	1.30	0.27	1.12	0.75	67.56
	CS	0.96	0.82	0.69	1.07	0.15	0.76	0.58	
	RC	1.18	1.07	1.14	1.35	0.28	1.17	0.79	
SD6	AAS	1.13	1.03	1.09	1.30	0.27	1.12	0.75	63.03
	CS	0.96	0.82	0.69	1.07	0.15	0.76	0.58	
	RC	1.18	1.07	1.14	1.35	0.28	1.17	0.79	

▲ Shoulder markers indicate restricted amino acids.

AAS, the amino acid score; CS, chemical score; RC, amino acid ratio coefficient; EAAI, essential amino acid index.

Apart from Met and Cys, the AAS and CS of the essential amino acids in SDs were greater than 1 and 0.6, respectively, indicating that the EAA is relatively well-balanced, which could provide a high-quality, comprehensive protein. The EAAI is a quantitative indicator of how well the measured essential amino acid content matches the standard protein content, with a value less than 75 indicating that the food is an inadequate source of protein (39). Additionally, the EAAI is a quantitative parameter that quantifies the relationship between the measured basic AA content and the standard protein content. Despite low protein content in SDs, the EAAI value indicated that SD3, SD1, SD4, and SD2 were still used as high-quality protein sources to meet human AA requirements.

### Mineral analysis

Minerals are one of the six essential nutrients that play a key role in maintaining the body’s metabolism and osmotic pressure balance (40). Table 7 summarizes the mineral contents of six SDs. Seven mineral elements were identified (Na, K, Mg, Ca, Fe, Zn, and Se). Furthermore, the Na content was significantly higher in the SD1, SD3, and SD6 groups than in the SD2, SD4, and SD5 groups (*P* < 0.05), which was consistent with the conductivity results in SDs. According to the United States daily recommended sodium intake of 2,400 mg, five tablespoons (one tablespoon is equal to 13 mg) of SD contained between 126.1 and 358.15 mg of Na, which was equal to 5.25–14.92% of the United States daily recommended daily 2,400 mg sodium allowance. Likewise, K is a key element that can aid muscle contraction and can alleviate hypertension caused by a high-Na diet (40). The K content in SD3 was significantly higher than that of the other groups (*P*°<°0.05). SD4 has 2–4 times more Mg than the other groups. Moreover, high Ca and Fe intake can help in promoting bone development and in improving iron deficiency anemia, and SD4 had the highest Ca and Fe contents (41). As these elements have similar physical and chemical properties, their biological functions are antagonistic, and this antagonism occurs when the Zn/Fe ratio is greater than one (42). Apart from the SD5 group, the zinc-to-iron ratio in SDs was acceptable. SDs had a higher mineral content and composition than some commercially available sauces and ketchup (43). SD is a good source of minerals, particularly SD4.

**TABLE 7:** Comparison of the mineral content in different sweet salad dressings.

	SD1	SD2	SD3	SD4	SD5	SD6
Na (mg/g)	4.56 ± 0.95^a^	2.50 ± 0.09^c^	4.14 ± 0.20^ab^	2.23 ± 0.29^c^	2.66 ± 1.15^c^	3.33 ± 0.01^b^
K (mg/kg)	54.45 ± 6.54^c^	21.96 ± 0.50^d^	88.53 ± 0.63^a^	15.91 ± 1.17^d^	64.14 ± 9.15^b^	71.27 ± 0.44^b^
Mg (mg/kg)	15.27 ± 3.64^c^	21.29 ± 3.38b^c^	18.19 ± 1.40^bc^	61.51 ± 1.69^a^	26.35 ± 1.75^b^	18.08 ± 5.41^bc^
Ca (mg/kg)	11.99 ± 1.05^a^	9.85 ± 2.62^a^	5.64 ± 1.17^b^	24.42 ± 0.38^a^	2.86 ± 2.46^b^	3.98 ± 1.34^b^
Fe (mg/kg)	12.18 ± 10.68^c^	7.32 ± 1.10^c^	40.11 ± 21.15^b^	56.44 ± 20.29^a^	4.44 ± 3.27^c^	5.45 ± 2.94^c^
Zn (mg/kg)	1.22 ± 0.52^b^	1.33 ± 0.33^b^	2.56 ± 2.63^b^	1.70 ± 0.60^b^	25.03 ± 5.93^a^	1.60 ± 0.09^b^
Se (mg/kg)	N.D.	0.02 ± 0.01	0.02 ± 0.01	0.03 ± 0.01	N.D.	0.03 0.00

Data are presented as mean ± SEM (n = 5). Different letters denote significant differences at P < 0.05 determined by ANOVA (Duncan’s test).

**FIGURE 1:**
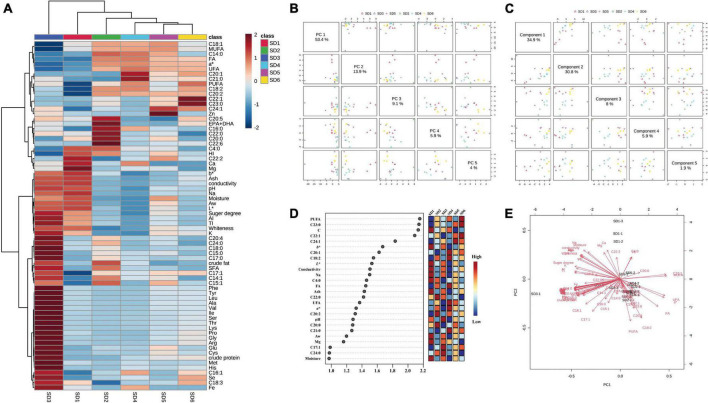
Analyses of principal components and partial least squares. **(A)** Ingredients heat map, **(B)** principal component analysis plot, **(C)** partial least squares analysis plot, **(D)** VIP values, and **(E)** salad dressings and nutrient content biplot model analysis. Thr, threonine; Val, valine; Ile, isoleucine; Leu, leucine; Lys, lysine; Met, methionine; Cys, cysteine; Phe, phenylalanine; Tyr, tyrosine; EAAI, essential amino acid index.

Principal Component Analysis (PCA) is a statistical analysis that reduces the dimensionality of a data set by linear transformation and performs a comprehensive analysis of the sample (44). Correlation analysis is a straightforward analytical technique for determining the relationship between two sets of quantitative data. The analysis may include the existence of a relationship between variables as well as the strength of that relationship (45). As illustrated in Figure 1, PCA and partial least squares analysis of the six SDs revealed that they were similar in composition and could be classified into five hierarchical categories. The SD3 was negatively correlated with C18:2, C18:1, UFA, and a* values and positively correlated with K, Fe, and crude protein contents, which differed from the other SDs.

## Conclusion

Salad dressings have gained popularity as a condiment because of their pleasant flavor. By examining and comparing the nutritional composition (physical properties, proximate composition, amino acids, fatty acids, and minerals) of six different sweet commercially available SDs in the Chinese market, the study established that SDs were within the range of acceptable nutritional values. The EAAI of SD3, SD1, SD2, and SD4 were found to be higher than FAO/WHO standards (EAAI°>°75) and to be high-quality protein sources. SD4 was an excellent source of EPA, DHA, and minerals (such as Mg, Ca, and Fe). Dietary intake of SD4 and SD6 appeared to be a better choice for reducing cardiovascular disease. The data from this study can provide basic parameters for determining dietary regimens for people with different food needs. In summary, SDs are an interesting source of bioactive compounds when consumed in moderation, and functional salad dressings for specific consumers could be developed in the future.

## Data availability statement

The original contributions presented in this study are included in the article/supplementary material, further inquiries can be directed to the corresponding authors.

## Author contributions

MY and MC: conceptualization, methodology, software, validation, and formal analysis. RM, TY, YX, and NT: investigation, resources, and data curation. XW: writing—review and editing. All authors contributed to the article and approved the submitted version.
